# Organ Allocation for Liver Transplantation According to the Public Opinion

**DOI:** 10.5812/hepatmon.6183

**Published:** 2012-08-20

**Authors:** Ahmad Danesh, Saharnaz Nedjat, Fariba Asghari, Ali Jafarian, Akbar Fotouhi

**Affiliations:** 1 Department of Epidemiology and Biostatistics, School of Public Health, Tehran University of Medical Sciences, Tehran, IR Iran; 2 Knowledge Utilization Research Center, Tehran University of Medical Sciences, Tehran, IR Iran; 3 Medical Ethics and History of Medicine Research Center, Tehran University of Medical Sciences, Tehran, IR Iran; 4 Hepatobiliary and Liver Transplantation Research Center (Imam Khomeini Hospital), School of Medicine, Tehran University of Medical Sciences, Tehran, IR Iran

**Keywords:** Liver Transplantation, Resource Allocation, Decision Making, Public Opinion

## Abstract

**Background:**

Although liver transplantation is the last resort for treating end stage liver diseases, this medical procedure is not available for all needful patients because of inadequate organ supply. Therefore, guidelines have been developed by medical experts to regulate the process. Some professionals believe that medical criteria are inadequate for organ allocation in all situations and may not secure fairness of organ allocation.

**Objectives:**

The current study has been designed to identify decision criteria about allocation of donated liver to potential recipients from public points of view.

**Patients and Methods:**

This is a qualitative study that was conducted through individual interviews and Focus Group Discussions. Individual interviews were conducted among patients’ companions and nurses in one of the two liver transplant centers in Iran. Group discussions were conducted among groups of ordinary people who had not dealt previously with the subject. Data was analyzed by Thematic Analysis method.

**Results:**

Most of the participants in this study believe that in equal medical conditions, some individual and societal criteria could be used to prioritize patients for receiving donated livers. The criteria include psychological acceptance, ability to pay post-operative care costs, being breadwinner of the family, family support, being socially valued, ability to be instructed, lack of mental disorders, young age of the recipient, being on waiting list for a long time, lack of patient’s role in causing the illness, first time transplant recipient, critical medical condition, high success rate of transplantation, lack of concurrent medical illnesses, not being an inmate at the time of receiving transplant, and bearing Iranian nationality.

**Conclusions:**

Taking public opinion into consideration may smooth the process of organ allocation to needful patients with equal medical conditions. It seems that considering these viewpoints in drafting organ allocation guidelines may increase confidence of the society to the equity of organ allocation in the country. This strategy may also persuade people to donate organs particularly after death.

## 1. Background

Fair allocation of scarce medical resources, such as donated organs, is one of the main challenges in the public health system (1, 2). Although organ transplantation has expanded due to progress in surgical techniques and using immunosuppressive medications, there is no balance between number of patients who need organ transplantation and available resources (3, 4, 5). The reason of imbalance relates mostly to shortage of donated organs and the increase in demand for receiving transplantation (6). This has resulted in need to select the recipients from a pool of eligible patients to allocate donated organs. In this view, medical experts in organ transplantation have tried to overcome challenges of recipient selection by presenting some guidelines and frameworks (7, 8, 9). The models given in some of these guidelines and frameworks are based on biological elements to reflect severity of illness and medical urgency of transplantation (10, 11). In many countries such criteria are acceptable to medical society and are being implemented for liver organ allocation. However, some surveys have shown that these criteria may not reflect the public opinions having concerns about fairness of allocating donated organs (12, 13). Although decision criteria that are attractive to the public may contain low or no medical value, attention to those criteria may assure the equity in distributing scarce resources among needful individuals, a pledge that is appealing to the public, especially to organ donors (14, 15). Unlike experts of organ transplantation who value to effectiveness of transplant operation, public polls of ordinary people are more likely willing to trade – off this effectiveness with personal and social criteria such as the patient’s age and role in his/her illness (16, 17). Considering all these elements, we are always facing with some questions: Is the selected recipient the best candidate for receiving transplantation? Does the selected recipient have higher priority to those who are in waiting list and are in need to organ transplant and its resultant benefits? Referring to the public opinion may raise and recommend a solution which is based on a theory that decision criteria given by the public usually embrace common values and beliefs in the society that can be used as a safeguard for equity and fairness of organ allocation.

## 2. Objectives

Given the facts that hepatic transplantation is yet a costly procedure with vital importance, and also scarcity of donated-liver resources liver transplantation was chosen as the subject of this study. Therefore, this study aimed to respond to the following question: “What are public-based decision making criteria to select recipients of liver transplantation according to Iranian opinions?”

## 3. Patients and Methods

### 3.1. Study Subjects

This is a qualitative study that was conducted through individual interviews and Focus Group Discussions (FGD). Purposeful sampling was used to choose potential participants. They were categorized into three groups according to their approach to liver transplantation services: 1) Recipients of the service (patients’ companions at the Imam Khomeini liver transplantation clinic at the time of registration); 2) Service providing personnel (nurses working at the liver transplantation ward); and 3) A sample group of ordinary people who had not dealt previously with the subject (a group of students studying at Tehran university of medical sciences and a group of employees working in a semi-private office). Target groups were selected in a way that a wide range of answers to question of the subject could be collected (characteristics of respondents are given in Table 1). Data were collected from patients’ companions and service providers through in-depth individual interviews, and from sample group by FGD. Data collection was finalized after saturation of responses by conducting twelve in-depth individual interviews and two FGDs with two separate groups of five ordinary people who had not dealt previously with the subject.

**Table 1:** Characteristics of the Respondents

	**Recipient of the Service**	**Service Providing Personnel**	**Groups of Ordinary People**
** Gender, No. (%) **			
Male	2 (33.3)	0 a	6 (60)
Female	4 (66.7)	6 (100)	4 (40)
** Age, y, mean ± SD **	33 ± 6.5	32.3 ± 4.6	36.9 ± 6.2
** Education, No. **			
Illiterate	0	0	0
Primary school	2	0	0
Guidance school	0	0	0
High school	2	0	0
University degree	2	6	10
** Marital status, No. **			
Single	2	2	5
Married	3	4	5
Divorce	1	0	0
Widow/widower	0	0	0

^a^Nurses working at Imam Khomeini liver transplantation center were all female

### 3.2. Data Analysis

All interviews were tape recorded and transcripts were thoroughly reviewed to elicit interviewees’ decision criteria. Expressed criteria by the interviewees were analyzed by Thematic Analysis method.

### 3.3. Ethical Consideration

Tehran University of Medical Sciences research proposal and ethical committees approved the study. A verbal consent on record of interview was obtained from each participant . In presented results, all measures were taken to avoid identity disclosure of interviewees.

## 4. Results

Based on the analysis of interviewees’ responses three categories of criteria were elicited: those criteria related to personal characteristics, to family or society, and to medical condition of the recipients. In each category, based on interviewees’ expressions, themes denoted interviewees’ preferred criteria to allocate donated liver were identified (Table 2). In order to better elaboration of responders’ views, some of their expressions are presented in italic throughout this section.

**Table 2:** The Groups of Criteria and elicited Themes Given by the Interviewers

**Category**	**Theme**
** Criteria related to personal characteristics **	
	Young age of the recipient
	Psychological acceptance
	Lack of mental disorders
	Ability to be instructed
	Lack of patient’s role in causing the illness
	Not being an inmate at the time of receiving transplant
** Criteria related to family or society **	
	Ability to pay post-operation care costs
	Family support
	Being socially valuable
	Being breadwinner of the family
	Bearing Iranian nationality
** Criteria related to medical condition of the recipient **	
	Critical medical condition
	First time transplant recipients (versus re-transplant candidates)
	Being on waiting list for a long time
	Lack of concurrent medical illnesses
	High success rate of transplantation

### 4.1. Criteria Related to Personal Characteristics of the Recipient

#### 4.1.1. Age

Age was a selection criterion to which was referred by almost all participants. In view of patients’ companions and service providers, younger individuals should be furnished by transplantation priority because of possessing higher probability of success rate, life expectancy, more tendencies to survive, and finally their precedence over old individuals given by the society.

“If people were told that a 25-year-old person died because the liver which might be donated to him/her was transplanted to a 75-year-old guy, an emotionally reaction may occur among our society and all would get upset and complain: why didn’t you substitute the 25-year-old patient for transplantation?”

The group of people who had not dealt previously with the subject also placed younger patients in priority due to the fact that they have the right to live longer and the more chance to play their role within the family and society. However, one of the interviewed personnel insisted on equal rights for all age groups to receive transplantation.

“In my opinion age is not really a criterion because all individuals have the right to live.”

Another respondent from the group also expressed that the older people could be better candidates for transplantation due to their responsibilities against the family.

#### 4.1.2. Psychological Acceptance

In view of patients’ companions, lack of psychological acceptance may result in fear about treatment outcome and its side effects, which eventually causes the doubt in person about transplantation. In contrast, psychological acceptance although not accompanied by an expectation of good result may encourage the patient to continue the treatment.

“As an example, my brother might receive (corneal) transplantation long time ago. He was not prepared for the operation at that time because of the fear that nothing would happen to him positively after the operation. When he got ready, he received it. Although more than two years have passed since his operation and no acceptable result has occurred, he has got along with the operation without problem. We may feel sad and worried, but he doesn’t at all”.

#### 4.1.3. Lack of Mental Disorders

In view of service providers and the people who had not dealt previously with the subject, “lack of mental disorders” such as depression was another defining criterion to prioritize patients. In fact, lack of depression endorses patient’s readiness to receive transplantation.

#### 4.1.4. Ability to be instructed

Being ready for compliance with postoperative medical orders was another criterion that was expressed by service providers.

#### 4.1.5. Lack of Patient’s Role in Causing the Illness

This brought about ideas both for and against this criterion. Among those who were in favor of this approach, in particular the people who had not dealt previously with the subject, it could be referred to the possibility of recommencing risky behavior after the operation that was more common among those who took risky behavior intentionally and continuously. Service providers believed that such patients were always suspected to return back to their previous risky behaviors and though were not suitable candidates of transplantation. In addition, due to the importance of drug abuse (as a risky behavior) to organ donors, risky behavior should be considered as a criterion for prioritizing patients.

“The one who donates his/her liver certainly likes to donate it to someone who really needs the organ but not to the one who doesn’t take care about him/herself and abuses drugs or exhibits other similar manners. I personally don’t see any priority for them”.

One service provider believed that because of high public awareness about AIDS and hepatitis B, no excuse would be acceptable about the lack of knowledge about these diseases and their respective ways of transmission. In addition, some socially unacceptable behaviors that can put individuals at risk of acquiring AIDS and hepatitis B can cause these individuals ineligible for transplantation even though they do not know about the ways of transmission.

“Now it can be said that the people are informed. Even if we think that the person was not aware of it, our culture considers the behavior as disgusting”.

In regard to the opposing views for considering this criterion in prioritizing recipients, it can be referred to unintentional and accidental risky behavior in some patients that was expressed by the people who had not dealt previously with the subject. One of the respondents in this group believed that the intention of making money in some individuals with risky behaviors (such as sex workers) can justify their behaviors. Therefore, they may be considered equally eligible to receive transplantation. to survive

#### 4.1.6. Not Being an Inmate at the Time of Receiving Transplant

In regard to legal conviction and being an inmate, there were ideas for and against it. According to the service providers, always there was a negative attitude towards past history of incarceration but due to the variety of reasons for conviction, it cannot be an appropriate criterion to prioritize patients. However, being an inmate at the time of receiving transplant could be considered as an important criterion for prioritization. This is mostly due to lack of easy access to post-operative medical care and its high costs. The people who had not dealt previously with the subject expressed that criminal history such as murder and theft, which resulted in conviction and punitive consequences, can make the recipient ineligible.

“Just think of a prisoner with a past history of conviction that has committed murder and several burglaries, now needs liver transplantation. In my opinion this person should not be considered for receiving transplant”.

### 4.2. Criteria Related to Family or Society

#### 4.2.1. Ability to Pay for Post-Operation Care Costs

Being rich enough to compensate post-operation medical care is one of the criteria that most of participants acknowledged. In view of some patients’ companions and the people who had not dealt previously with the subject, although transplantation is free in the country, post-operation medical care costs could be much more expensive than the operation itself due to the lack of or insufficient insurance coverage for recipients. On the other hand, one of the participants in this group stated that the wealth may facilitate post-operation medical care, but this criterion may cause inequity in allocating donated organs to the patients.

“It is not right that rich people enjoy more but poorer be left alone until the death. This is not fair at all”.

#### 4.2.2. Family Support

The patients’ companions believed that family support could be financial, psychological, or providing the patient with peace at home after the operation. In view of service providers, family support is among important decision-making criteria to allocate donated organ to the patients because of its effect on following up medical care after the operation, compensating treatment costs, and providing living standards at home.

#### 4.2.3. Being Socially Valued

In view of service providers the value of an individual from two perspectives was important: economical and job experiences. Highlighting this criterion is rooted in financial needs of patient’s relatives and also in their social role for transferring experiences to their successors. In fact, these needs associate with family economy and patient’s effect on the society. The people who had not dealt previously with the subject also had views similar to those of service providers.

“The value depends on the positive effects of people which don’t mean that the patients should occupy high ranked professionals. We just need to look at the patient and see how influential this person is in the society as in performance and attitudes”.

#### 4.2.4. Being Breadwinner of the Family

The group of service providers similar to the people who had not dealt previously with the subject considered the being breadwinner of the family a valuable criterion for prioritization due to dependence of the family on the patient’s income and also difficulty in managing expenses after losing their breadwinner.

“Since some of these patients are breadwinners, if they don’t receive donated organ and keep suffering from the illness, the family cannot (even) make ends meet”.

#### 4.2.5. Iranian Nationality

The service providers mentioned that the current national organ allocation guidelines emphasize that the collected organs from Iranian donors with brain death should only be allocated to Iranians. The group believed that this strategy, in addition to addressing needs of Iranian patients, to some considerable extent can prevent donated organs from being abused. However, after receiving permission from donor’s family, it is possible to disregard this criterion for organ allocation. The people who had not dealt previosly with the subject believed the life of any individual as a national asset and is in the possession of the country of origin. Therefore, they considered allocation of donated liver to the same nationality as a criterion for prioritization.

### 4.3. Criteria Related To Medical Condition of the Recipient

#### 4.3.1. Critical Medical Condition

In spite of emphasizing nonmedical criteria for prioritizing patients at the beginning of the interviews, most interviewees considered critical condition of the patient as an important criterion. Patients’ companions presented high probability of death, not having enough time for receiving another transplantation, and dismal medical condition of the patient as the reasons for considering this criterion.

#### 4.3.2. First Time Transplant Recipient

In regard to this indicator it seemed that service providers did not consider re-transplant candidates as suitable candidates compared to the first time transplant recipients because of higher costs of post-operation care and their unstable situation. In their view, likely noncompliance of re-transplant patients with physician orders in the first attempt that caused rejection was one of reasons for considering them as unsuitable candidates. In addition, organ donation to re-transplant candidates deprives first time recipients from chance of transplantation which has never occurred for this group. The people who had not dealt previously with the subject believed that if the reason for organ rejection was medical malpractice, the patients should be high ranked to receive organ otherwise transplantation should not be allocated for them.

#### 4.3.3. Being on Waiting List for a Long Time

Service provides and the people who had not dealt previously with the subject believed that waiting time for receiving liver transplantation could be considered as a criterion for prioritization. One of the people who had not dealt with the subject even stated that this criterion could be the only one that should be employed.

“In my opinion, whenever one of my family members may get involved, I can accept nothing but this (i.e. time on waiting list). I personally do accept that (i.e. time on waiting list) but not age, occupation, number of relatives, children, or any other factors”.

#### 4.3.4. High Success Rate of Transplantation

In view of service providers, the operation usually should be performed on a patient that can get the best result after the operation. The people who had not dealt previously with the subject proposed quite similar opinion.

“Even in a serious medical condition of a patient, if we know that the operation may not go well, in my opinion, the one with a better medical condition but a better treatment outcome is in priority”.

Finally, there are some factors to be pointed out that from the interviewees’ points of view were not significant to prioritize the patients. These factors include education, gender, religion, ethnicity, and race. 

## 5. Discussion 

In the current study that was conducted to define criteria for liver organ allocation to patients, interviewees referred to personal, familial, social, and medical criteria. Although some of these criteria were in the list of contraindications to liver transplantation (i.e. lack of patient’s role in causing the illness rooted in the active substance abuse, family support rooted in the lack of psychosocial support, and ability to be instructed rooted in the inability to comply with medical illness), the criteria which were mostly expressed by the participants were not currently considered for organ allocation (18, 19). Such expressed thoughts by interviewees can imply this idea that equal to medical situations, people would prefer to use personal, familial, and social criteria for selecting suitable recipients; a point that is not mentioned in the current guidelines for organ allocation. In this study, the important matter which was expressed by the participants was the severity of patient’s medical condition as the first criteria for selecting recipient. Severity of patient’s medical situation is the criterion that is always given along with other criteria such as young age of recipients and long waiting time for receiving organ transplantation (16, 17). This has highlighted the importance of medical criteria for the public as the first criterion for allocating donated organs to those who are in need to organ transplantation.

Although there are ideas for and against criteria related to patient’s role in their medical illness or past history of imprisonment as one of the criteria for prioritizing patients, interviewees emphasized that intentional risky behaviors in the past that can put patients at risk for liver disease or imprisonment at the time of organ allocation should be considered to prioritize patients. These criteria were previously expressed by the public and medical experts in other studies (20). Participants also indicated some criteria such as ability to pay post-operative care costs (to comply with physician’s orders) and being socially valued(to strengthen economy of the family and society) that are in contrast with current guidelines in allocating donated organs to patients (21). In addition, some of the interviewees mentioned equality in allocation of the resources that was based on prioritizing the first time transplant recipients over the re-transplant candidates; a criterion that was referred to in previous studies (22). Although these criteria seem to be acceptable to the public for prioritizing patients, measuring some of them as organ allocation criteria does not seem to be easy. Among the participants there were still some people who believed in pure medical criteria for organ allocation, and among above mentioned criteria they believed in being on waiting list for a long time as the only effective criteria for selecting transplant recipients. Another important and attractive criterion was Iranian nationality of the recipients as a criterion for prioritization. This idea is in line with the current national law in order to prevent organ trafficking. According to the national guidelines, refugees will be supported financially and their post transplantation care services will be covered after they receive transplantation from a national mate; A regulation in Iran that may not exist in many countries in the region (23). The interviewees also pointed out a few criteria which were novel to investigators and not mentioned in previous studies. These criteria consist of psychological acceptance of transplantation and ability to be instructed for post-surgery care (the latter could be rooted in the inability to comply with medical illness). In view of the participants, family support could play substantial role in this regard. In addition, interviewees believed that some of personal and social criteria such as education, sex, ethnicity, race, and religion were not judged as defining criteria to select recipients and should not be employed to prioritize the patients. It is important to mention that the objective of this study was defined as collection a range of public opinions on non-medical criteria in allocation of donated livers to patients. It was not meant to prioritize the criteria based on their importance. Furthermore, because of applying purposeful sampling, findings of this study expressed by a few participants should not be generalized to the public and be used as selection criteria for liver recipients. In spite of all these limitations, noticeable characteristics in all qualitative studies, it seems that the study was able to open a window into people thought in the area of allocating organs to patients. This is happening in an era that the possibility of using organ transplantation has raised some other issues such as increase in financial resource shortage for transplantation, decrease in services to recipients, and most importantly shortage of organs used for transplantation. As a situation that each competent country for organ transplantation like Iran will face with after the law of “organ transplantation and brain death” law is passed (24, 25, 26, 27, 28). Although this study tried to explore a range of personal, familial, and societal criteria by referring to participants’ views, interviewees emphasized that the criteria should collectively be taken into account in order to select an eligible recipient. Accordingly, it is required that the importance of each of these criteria be measured against other criteria and the recipient be selected by considering all these elements. In order to reach this goal it is recommended to identify the value of those criteria based on quantitative studies. If so, scientific use of these criteria in the future of decision making applied by the physicians will be promising.
